# Phylogenetic and phenotypic characterization of *Fusarium oxysporum* f. sp. *niveum* isolates from Florida-grown watermelon

**DOI:** 10.1371/journal.pone.0248364

**Published:** 2021-03-25

**Authors:** James C. Fulton, B. Sajeewa Amaradasa, Tülin S. Ertek, Fanny B. Iriarte, Tatiana Sanchez, Pingsheng Ji, Mathews L. Paret, Owen Hudson, Md. Emran Ali, Nicholas S. Dufault

**Affiliations:** 1 Department of Plant Pathology, University of Florida, Gainesville, Florida, United State of America; 2 The Institute for Advanced Learning and Research, Danville, Virginia, United State of America; 3 Zirai Mücadele Merkez Araştırma Enstitüsü, Ankara, Turkey; 4 North Florida Research and Education Center, University of Florida, Quincy, Florida, United State of America; 5 University of Florida Institute of Food and Agricultural Sciences, Alachua County, Florida, United State of America; 6 Department of Plant Pathology, University of Georgia, Tifton, Georgia, United State of America; Franklin & Marshall College, UNITED STATES

## Abstract

Fusarium wilt of watermelon (*Citrullus lanatus*) caused by *Fusarium oxysporum* f. sp. *niveum* (Fon), has become an increasing concern of farmers in the southeastern USA, especially in Florida. Management of this disease, most often through the use of resistant cultivars and crop rotation, requires an accurate understanding of an area’s pathogen population structure and phenotypic characteristics. This study improved the understanding of the state’s pathogen population by completing multilocus sequence analysis (MLSA) of two housekeeping genes (BT and TEF) and two loci (ITS and IGS), aggressiveness and race-determining bioassays on 72 isolates collected between 2011 and 2015 from major watermelon production areas in North, Central, and South Florida. Multilocus sequence analysis (MLSA) failed to group race 3 isolates into a single large clade; moreover, clade membership was not apparently correlated with aggressiveness (which varied both within and between clades), and only slightly with sampling location. The failure of multilocus sequence analysis using four highly conserved housekeeping genes and loci to clearly group and delineate known Fon races provides justification for future whole genome sequencing efforts whose more robust genomic comparisons will provide higher resolution of intra-species genetic distinctions. Consequently, these results suggest that identification of Fon isolates by race determination alone may fail to detect economically important phenotypic characteristics such as aggressiveness leading to inaccurate risk assessment.

## Introduction

Watermelon (*Citrullus lanatus*) production is economically important to the southern United States including Florida, Texas, and Georgia as well as other countries throughout the world [1–3]. Biotic constraints such as the vascular wilt disease caused by *Fusarium oxysporum* f.sp. *niveum* [4] significantly limits yield potential [5, 6]. This ubiquitous soil-borne fungal pathogen has been reported in watermelon fields throughout the world, yet within the watermelon production areas of the United States, race 2 appears to be the dominant concern due to its widespread distribution and the lack of resistant watermelon cultivars [7, 8].

Fusarium wilt, caused by the hemibiotrophic fungus *Fusarium oxysporum*, often appears as seedling damping-off, or graying foliage, which precedes widespread chlorosis or leaf necrosis, before eventual wilt and senescence. A single runner vine often shows wilting symptoms leading to unilateral necrosis while the rest of the plant is asymptomatic [8]. Under wet environmental conditions, rosette hyphae can be observed erupting from necrotic plant tissue [9]. These common wilting symptoms are the result of complex host pathogen interactions which begin when resting chlamydospores sense root exudate from susceptible seedlings, extend and attach hyphae to root surfaces. During compatible interactions, hyphae penetrate plant roots and colonize the plant via its vascular system [10]. As response to the expanding colonization of its vascular infrastructure, the host plant produces tyloses, or outgrowths of parenchyma cells, to restrict and confine hyphal infiltration to already infected vascular tissue [11]. However, despite these common symptoms, there is significant variation depending on race, host x race interactions, inoculum quantity, and environmental conditions including seasonal temperature variations, so that the duration of infection prior to ultimate senescence may be several weeks, with variable levels of field incidence [12–15].

As suggested by disease symptom variability, particular genetic assemblages or races within the *forma specialis* niveum, have emerged due to selective pressure resulting from field deployment of resistant cultivars [16]. Currently, four races (0, 1, 2, and 3) are recognized and identified by their pathogenicity to standard test cultivars [12]. However, this apparent genetic variability exists despite Fon lacking any identified sexual stage and instead produces two asexual spore structures designated as micro and macroconidia [14]. Microconidia, produced in an abundant quantity, are oval-shaped while macroconidia are fusiform, deposit 2–4 septa, and occasionally develop structures called sporodochia [17]. Another common structure are chlamydospores which promote long-term survival (6–10 years) for pathogen propagules disseminated under unfavorable environmental conditions [18]. For example, chlamydospores may reside in fallow soil for several growing seasons until susceptible cultivars are sown. In addition to its survivability, the pathogen may also be disseminated by severe weather, the movement of contaminated materials (e.g. heavy equipment, soil, clothing, etc.), and shipment of latently infected transplants to virgin fields [17].

Given the ability of Fon to survive in soil for several years as well as the ease with which it disseminates once introduced to a new area, management strategies emphasize exclusion through the use of clean seed and planting stocks; however, once inoculum is present in a field, management strategies may include host resistance, grafting, and crop rotation [17]. Unfortunately, many of these previously adequate solutions have become unsatisfactory as new environmental regulations restrict the application of certain chemical classes such as methyl bromide (MeBr); consumer preference encouraging the cultivation of Fon susceptible triploid (seedless) watermelon cultivars; and new races have emerged that are pathogenic in previously resistant cultivars [5, 19, 20]. As a result of these new conditions, long-term rotations spanning 6–10 years with non-host species is increasingly ineffective in limiting the economic impact of Fon [18].

Recently, a new strain, race 3, was identified in Maryland which poses a greater threat to watermelon cultivation as it is able to cause disease in previously resistant cultivars [12]. This new race appears to exist in locations outside the area of first detection and is now present in several counties in the south-eastern United States [21, 22]. Hypotheses explaining the emergence of race 3 as well as other *Fusarium oxysporum* races have proposed that the presence of numerous transposons in the genome, which may be under less stabilizing selection pressure, are more likely to mutate and thus produce new pathogen effectors [23, 24]; additionally, horizontal gene transfer, of pathogenicity-related genes and entire chromosomes has been demonstrated in the laboratory [25, 26]. Both hypotheses have received support as past molecular analyses of *F*. *oxysporum* formae speciales have revealed divergent lineages with multiple independent origins [27–30], and multilocus gene analysis has also suggested biogeographic structure for some others [29, 31]. Thus, determining whether the observation of race 3 in new locations resulted from their introduction or novel creation is challenging. Contributing to this difficulty is the possible obfuscation resulting from the categorization of strains into one of four groups (e.g. races 0, 1, 2, or 3) as the differential cultivar method is dependent on the presentation of plant symptoms, which are affected by additional factors such as host environment, inoculum level, and isolate aggressiveness [32].

The objectives of this study were to (1) describe the genetic and phenotypic (e.g. aggressiveness and race type) diversity of Fon isolates collected from Florida watermelon fields over a four-year period, (2) identify the degree to which geography contributes to the genetic structure of Florida Fon isolates, and (3) characterize any correlation between geography of sampled locations and phenotype (e.g. aggressiveness and race) with sequenced loci.

## Materials and methods

### Isolate collection

A total of 72 *Fusarium* sp. isolates, including 2 isolates from diseased squash (*Cucurbita* sp.), were collected from watermelon fields located in 11 counties in Florida and grouped into three geographic populations including a North population (Pop1, 44 isolates): Alachua, Gilchrist, Madison, Suwannee, and Jackson counties, Central population (Pop2, 16 isolates): Levy, Marion, Putnam, St. Johns, and Sumter counties, and a South population (Pop3, 11 isolates): Lee county (Table 1). Isolate 150331, previously identified as race 2, from Cook County, Georgia was also included and grouped into the North population. F*usarium sporotrichioides* (isolates PUF032 and NRRL 53434) and *F*. *solani* (isolates PUF006 and M113A) were included as phylogenetic outgroups. Isolates were either directly collected by authors or provided by University of Florida diagnostic laboratories located in satellite campuses throughout the state. Unique isolates came from separate plants and the sampling location was recorded. The diseased tissue was cut into slices and surface-sterilized with 0.6% sodium hypochlorite for 1 min, rinsed in water and transferred to quarter strength potato dextrose agar (qPDA). Pure cultures were obtained by transferring hyphae emerging from the sliced tissue to new qPDA plates. A single hyphal tip from each culture was then grown on an individual qPDA plate with inlaid filter paper pieces covering the majority of the media surface area. After three weeks, mycelia covered filter papers were dried thoroughly in a desiccator and stored at -80°C for later use.

**Table 1:** *Fusarium oxysporum* f. sp. *niveum* [4] isolates analyzed in this study. Fon host plant was watermelon unless otherwise indicated.

ID	Year	Location	Aggressiveness	Bioassay	Six6 (Positive/Negative)
150523	2015	Madison	Moderate	1	P
150514–1	2015	Alachua	Moderate	0	P
150328	2015	Lee	Moderate	3	N
150601–5	2015	Madison	Severe	3	P
150321	2015	Lee	Moderate	3	N
150319	2015	Lee	Severe	3	N
150602–1	2015	Madison	Severe	2/3	P
150601–4	2015	Madison	Severe	3	P
150515–5	2015	Suwannee	Moderate	2/3	N
140507-b	2014	Levy	Severe	0	P
150515–2	2015	Suwannee	Moderate	2	N
110407.1-1.B2.F9C1	2011	Alachua	Zero	0	N
140507-a	2014	Levy	Moderate	2	P
140508-b	2014	Alachua	Severe	0	P
140411	2014	Alachua	Severe	0	N
150320	2015	Lee	Severe	3	N
150514–3	2015	Alachua	Moderate	3	P
150416	2015	Madison	Severe	0	P
150524	2015	Madison	Severe	3	P
130507	2013	Alachua	Moderate	0	P
150525	2015	Madison	Severe	3	P
150526	2015	Madison	Severe	3	P
150527	2015	Marion	Severe	3	P
150515–3	2015	Suwannee	Severe	2/3	N
150516–2	2015	St. Johns	Zero	0	N
150601–1	2015	Madison	Severe	3	P
150601–2	2015	Madison	Severe	3	P
150601–3	2015	Madison	Severe	2	P
111006	2011	Marion	Zero	0	P
111017	2011	Putnam	Weak	0	P
150514–2	2015	Alachua	Severe	2/3	P
140506	2014	Levy	Moderate	2	N
140704	2014	Marion	Zero	0	N
150318	2015	Lee	Moderate	2	P
150322	2015	Lee	Moderate	3	P
150323	2015	Lee	Weak	2/3	P
150324	2015	Lee	Weak	3	P
150325	2015	Lee	Weak	3	P
150326	2015	Lee	Weak	3	N
150327	2015	Lee	Weak	3	P
150329	2015	Jackson	Weak	NON	P
150515–1	2015	Suwannee	Moderate	3	N
150330	2015	Jackson	Zero	NON	N
150408	2015	Levy	Moderate	3	P
150409	2015	Levy	Weak	3	P
150410	2015	Levy	Moderate	2/3	P
150411	2015	Gilchrist	Zero	NON	N
150412	2015	Gilchrist	Zero	3	N
150413	2015	Gilchrist	Zero	1	N
150414	2015	Gilchrist	Zero	3	N
150417	2015	Madison	Severe	2/3	P
150512	2015	Suwannee	Weak	3	P
150515–4	2015	Suwannee	Moderate	2	
150513	2015	Marion	Moderate	2/3	N
110407.1-1.B2	2011	Alachua	Zero	0	P
110407.1-1.B2.F9C2	2011	Alachua	Zero	0	P
110407.1-1.B2-F8	2011	Alachua	Zero	0	N
110407.1-1.B2-F8C	2011	Alachua	Zero	NON	N
110407.2–1	2011	Alachua	Zero	0	P
110407.3–1.1	2011	Alachua	Zero	0	N
130513	2013	Sumter	Moderate	0	N
110407.3–1.2	2011	Alachua	Zero	0	P
110407.3-1.B	2011	Alachua	Weak	NON	P
110407.3–2.2	2011	Alachua	Zero	0	P
110407.3-4.B1	2011	Alachua	Zero	0	N
110407.3-4.B2	2011	Alachua	Zero	0	P
110407.3-4.B3	2011	Alachua	Zero	0	P
111018–2	2011	Putnam	Zero	3	N
111018–4	2011	Putnam	Zero	0	N
140508-a	2014	Alachua	Severe	1	N
150516–1	2015	St. Johns	Zero	0	N
150331	2015	Cook*	Severe	2	N

*Cook Co, GA.

**Pathogen isolates were extracted from squash.

Severity categories includes zero, weak, moderate, severe.

The bioassay column indicates the isolate’s race (0–3) as determined by plant inoculation trials.

NON: non-pathogenic.

The Six6 column indicates whether the Six6 effector gene was amplified as shown by gel electrophoresis.

P/N indicates positive/negative PCR amplification.

### DNA extraction

Isolates were revived on qPDA plates laid with GelAir cellophane film (Bio-Rad Laboratories Inc., Hercules, CA) at room temperature for one week. The mycelia were scraped off the cellophane and 100 mg transferred to 1.5 ml Eppendorf tubes. The samples were lyophilized and DNA extracted using a DNeasy Plant Mini Kit (Qiagen Inc., Valencia, CA).

### DNA amplification and sequencing of loci

Molecular markers were chosen partially based on research by O’Donnell et al. (2009) [33], who showed that the translation elongation factor (TEF) gene and intergenic spacer region of rDNA (IGS) possess higher nucleotide diversity compared to other coding and non-coding genomic regions previously tested. Additionally, these were complemented by the internal transcribed spacer (ITS) locus and β-tubulin (BT) gene (Table 2). Partial TEF (~690 bp), BT (~600 bp), and ITS (~500 bp) genes and locus were amplified using primer pairs EF1/ EF2 [28], T1/ T22 [34], and ITS5/ITS4 [35] respectively. The IGS locus was amplified using three primer pairs: INL11/iNLr, NLa/CNSa, and CNS2/iCNS1 [33] and aligned together to form a single contig. The polymerase chain reaction (PCR) was performed as described in [34] with Phusion High-Fidelity DNA polymerase (New England BioLabs Inc., Ipswich, MA) in a thermocycler (Eppendorf, Hamburg, Germany). The following program was used for amplifying ITS and BT loci: 1 cycle of 150 s at 98°C; 32 cycles of 10 s at 98°C, 50 s at 52°C, and 55 s at 72°C; followed by 1 cycle of 10 min at 72°C and held at 4°C. The remaining primer pairs were given the same conditions except for the annealing temperature. The TEF primers, and iNL11/iNLr and CNS2/iCNS1 of IGS had an annealing temperature of 55°C while NLa/CNSa primer pair was amplified using an annealing temperature of 65°C. All PCR fragments were visualized and verified for correct size range by electrophoresis on 1.2% agarose gels stained with Sybr Green I Nucleic Acid Gel Stain (Invitrogen, Waltham, MA) using 1 x TAE buffer. The PCR products were purified using a Wizard SV Gel and PCR Clean-UP System kit (Promega Corporation, Madison, WI) and shipped to Eurofins Genomics LLC (Louisville, KY) for Sanger sequencing using both forward and reverse primers. The sequences of all isolates were deposited in GenBank (http://www.ncbi.nlm.nih.gov/) for reference. Accession numbers are shown in S1 Table. The GenBank accessions of outgroups were HQ165837 and HQ165863 (TEF); KP710625 and HQ141662 (BT); HQ165909 and HQ165935 (ITS); and HQ165873 and HQ165899 (IGS) for *F*. *solani* and *F*. *sporotrichioides*, respectively.

**Table 2:** The TEF, BT, and FonSix6 genes, and ITS and IGS loci described in this research were amplified using the following primers during polymerase chain reaction cycles with the listed thermocycler settings.

Gene/Locus	Sequence (5’-3’)	Gene/Locus product	Tm (°C)	Aligned fragment length (bp)	Reference
TEF	EF-1: ATGGGTAAGGA(A/G)GACAAGAC	Translation elongation factor (EF-1α)	55	~690	[28, 33]
	EF-2: GGA(G/A)GTACCAGT(G/C)ATCATGTT				
IGS	INL11/iNLr^1^: AGGCTTCGGCTTAGCGTCTTAG/ AATTCTACTTACCCTAGAGC	Nuclear ribosomal intergenic spacer region	^1^: 55	2500	[33]
	NLa/CNSa^2^: TCTAGGGTAGGC—GTTTGTC/ TCTCAT—TACCCTCCGAGACC		^2^: 65		
	CNS2/iCNS1^1^: AACTTCAATCGCCTCTCACG/ TTTCGCAGTGAGGTCGGCAG				
ITS	ITS5: GGAAGTAAAAGTCGTAACAAGG	Nuclear ribosomal internal transcribed spacer region	52	~500	[35]
	ITS4: TCCTCCGCTTATTGATATGC				
BT	T1: AACAT-GCGTGAGATTGTAAGT	β-tubulin	52	~600	[34]
	T22: TCTGGATGT-TGTTGGGAATCC				
Fon*SIX*6	Fon*SIX*6F: CGCTCTTATCGCATCAATCT Fon*SIX*6R: GGGTTGACTGAGGTCGTGGT	Secreted in xylem protein 6	59	~438	[11, 36]

The FONSIX6 gene was amplified due to previous research describing its utility in differentiating Fon races 0 and 1 from race 2 [11]. Niu et al. (2016) reported that the gene was present in races 0 and 1 while absent in race 2. However, Keinath et al. (2020) reported inconsistencies with this identification and suggested further demonstration of its efficacy to successfully distinguish Fon races [7]. The PCR primers and thermocycler settings described by Hudson et al., 2020 [36] were used for amplification; specifically, 1 cycle of 180 s at 94°C, 35 cycles of 30 s at 94°C, 30 s at 59°C, 60 s at 72°C, and finally 420 s at 72°C. Polymerase chain reactions (PCRs) contained 12.5 μL EconoTaq PLUS Green 2X Master Mix (Lucigen, Middleton, WI), 9.5 μL ddH2O, 1 μL of forward and reverse primers, and 1 μL of genomic DNA. 5 μL (20 ng DNA/μL) of each reaction were loaded into a 1% agarose gel with 4 μL of GelGreen 10,000x (Thomas Scientific, Swedesboro, NJ).

### Multilocus sequence analysis

After removing reference isolate UGA-Fon-30, *Fusarium sporotrichioides* isolates PUF032 and NRRL 53434, and *F*. *solani* isolates PUF006 and M113A, the forward and reverse sequences were aligned and consensus sequences were made using Geneious Version 9.1 [37]. Alignment of the sequences was completed using the MUSCLE algorithm [38] in MEGA 7 software for each locus [39]. The missing sequence data at each end of the alignment were trimmed to remove ambiguous regions and then phylogenetic trees were constructed. Phylogenetic analysis was done in MEGA 7 to generate a neighbor-joining (NJ) tree with Kimura-2-parameter (K2P), and a Maximum Likelihood [40] phylogeny using the nearest-neighbor interchange algorithm and general time-reversible (GTR) substitution model [39, 41]. To ascertain the integrity of clades, bootstrap tests were performed with 500 replicates.

### Haplotype network

Each unique concatenated sequence was defined as a haplotype and the number of unique haplotypes was calculated using the Sidier R package [42, 43]. The infinite sites model [44] was used to build the haplotype network in the Pegas package [45]. The haplotype network pools identical sequences into a single node or vertex and can be drawn proportionately to the number of sequences with the same haplotype. The isolates were color-coded according to their sampled location (subpopulations), aggressiveness, and year of collection to derive relationships with sequence data.

### Genetic diversity of *Fusarium* populations

Sequence data were uploaded to the R Apex package [46] and the genetic differentiation statistics, Nei’s G_ST_ [47], Hedrick’s Gʹʹ_ST_ [48, 49], and Jost’s D [50] for Fon populations were calculated using Mmod package [51]. The range of Nei’s G_ST_ can be restricted by allele diversity and therefore, interpretations can be difficult [52]. Further, G_ST_ is influenced by the number of alleles at each locus and the number of populations sampled [53]. Hedrick’s Gʹʹ_ST_ is a standardized measure calculated by dividing G_ST_ for a given locus by the maximum G_ST_ value based on the diversity of that locus [54]. Jost’s D is different from the previous two statistical methods since it uses the effective number of allelic variations among populations rather than heterozygosity for calculating diversity [55].

DnaSP v. 6.10.00 software [56] was used to calculate i) haplotype diversity (H): the probability that two randomly sampled haplotypes are different [47]; ii) nucleotide diversity (π): the average number of nucleotide differences per site between two sequences [47]; iii) gene flow: the exchange of isolates, genes, or DNA sequences between populations, which was determined by tallying the number of polymorphic sites [57, 58]; and iv) Tajima’s D: a test to determine if mutations are selectively neutral [59]. Tajima’s D statistic can also test whether sequences comply with the infinite-sites model [44], which assumes there are a large number of sites where mutations can occur and every mutation occurs on a new site, whereby only two different character states (mutation or wild state) per site can be present. The amount of variation originating from within individual *Fusarium* populations was compared to that portion of the variation between individual populations was evaluated by analysis of molecular variance (AMOVA) [60] using Pegas package of R program [45].

### Assessing aggressiveness and race type

Isolates were grown on 10-cm-diameter Petri plates having V8 (200 ml V8 juice, 15 g agar, 1% CaCo3, and 800 ml distilled water) and qPDA media for one week to generate conidia. Mycelia from these plates were scraped by adding 5 ml of sterile water per plate and transferred to a 50 ml conical centrifuge tube. The spore concentration of the solution was determined by a hemocytometer and final concentration adjusted to 1 x 10^6^ conidia/ml by adding water. Watermelon cultivars were sown and cultivated at the University of Florida. Later, twelve day old Fusarium wilt susceptible ‘Black Diamond’ watermelon seedlings were subjected to standard root dip inoculation [61] by suspending roots in 20 ml of the spore solution for 45 s. The control seedlings were root inoculated with water. Six seedlings were inoculated per Fon isolate or water control. Root inoculated seedlings were transplanted in 72-cell polystyrene foam trays with Professional Growing Mix potting media (Sun Grow Horticulture Canada Ltd. Seba Beach, Canada). The flats were kept in a greenhouse with an average daytime temperature of 27°C and nighttime temperature of 24°C, and a 14 h photoperiod. Seedlings were observed for Fusarium wilt incidence every week for 28 d. Isolates were grouped into four aggressiveness groups based on an ad hoc disease severity index: non-aggressive, weak, moderate, and severe, which corresponded to 0%, 1–32%, 33–67%, and 68–100% wilt symptoms on all the plants examined, respectively. The experiment was replicated four times. Simpson’s Index of Diversity (1-D), which accounts for variety and count frequency of object studied, was calculated for the levels of aggressiveness [62, 63]. Simpson’s index ranges from 0–1 and represents the probability that two isolates randomly selected from a sample are from different aggressiveness categories.

Race typing was conducted by root dip inoculation using six plants per each of the four watermelon differentials (Black Diamond, Charleston Grey, Calhoun Grey, Plant Introduction 296341-FR) with specific resistance as described by Zhou et al [12]; however, the susceptible variety ‘Sugar Baby’ was replaced with ‘Black Diamond’ in our experiment. Race assessment was then determined by observed disease incidence 28 d after inoculation. Cultivar differentials with disease incidence less than 33% were considered resistant while those with incidence greater than 33% were considered susceptible. For example, if all Black Diamond plants were symptomatic but none of the other cultivars were affected, then the isolate were identified as race 0. However, if all Black Diamond plants were symptomatic and 3 of the Charleston Grey plants were also symptomatic then the isolate would be classified as race 1. Races are determined in an incremental fashion with race 3 producing symptoms in all cultivars. Aggressiveness and race typing experimental results are included in S1 File.

## Results

### Genetic diversity and phylogenetic analysis

Descriptive statistics of aligned sequences are depicted in Table 3. The four-locus combined data set contained 54 unique haplotypes. The TEF region gave the highest percentage of segregating sites (126/580 = 21.7%) relative to 580 total sites. Gene flow estimates among *Fusarium* subpopulations were lowest for the IGS region (Nm = 0.83) and highest for the ITS region (Nm = 79.25). The coefficients of genetic differentiation statistics for tested Fon isolates are listed in Table 4. All Tajima’s D values were negative but only BT, ITS, and TEF regions showed statistical significance at *P* < 0.05.

**Table 3:** Polymorphism statistics derived from multiple sequence alignments of different loci analyzed.

Gene/locus region	Length	Parsimony informative sites	Segregating sites (S)	Number of haplotypes	Haplotype diversity	Nucleotide diversity (π)	Gene flow^a^
BT	457	11	25	21	0.659	0.0038	10.13
IGS	2454	110	191	33	0.916	0.0149	0.83
ITS	464	4	88	12	0.396	0.0073	79.25
TEF	580	25	126	13	0.681	0.0112	49.20
Combined Loci	3955	150	430	54	0.985	0.0122	1.26

^a^ Gene flow from N_ST_ with Jukes-Canter correction. [64].

**Table 4:** Genetic differentiation statistics and Tajima’s D neutrality test^a^.

Gene/Loci region	Nei’s G_ST_	Hedrick’s G′′_ST_	Jost’s D	Tajima’s D
BT	0.0090	0.0423	0.0292	-2.088*
IGS	0.0768	0.6933	0.6550	-0.436
ITS	0.0778	0.5384	0.4799	-2.853*
TEF	0.0344	0.2821	0.2437	-2.622*
Combined Loci	0.0219	0.9725	0.9716	-1.718

^a^ Asterisk (*) indicates statistical significance for Tajima’s D at *P* < 0.05.

**Fig 1:**
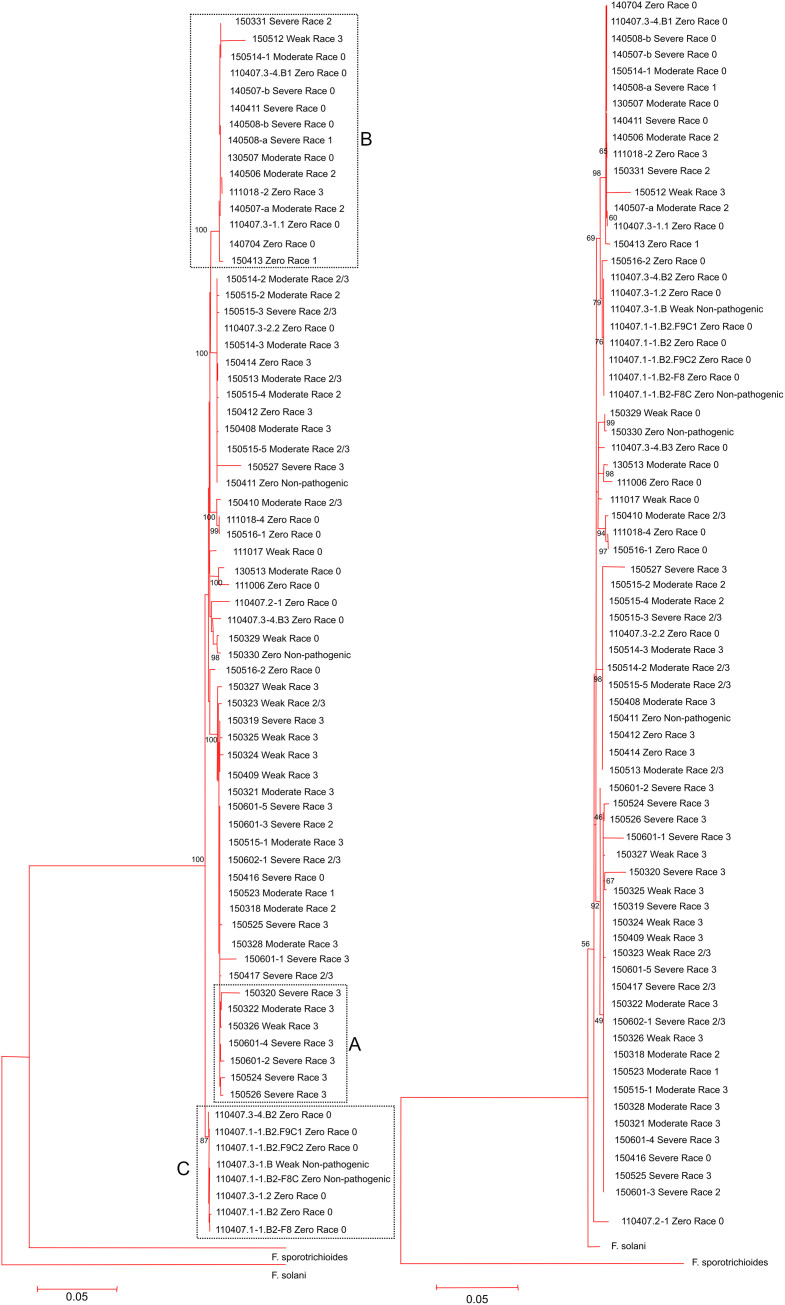
Molecular phylogenetic analysis from concatenated gene sequences. The evolutionary history was inferred by using a (A) Neighbor-joining (NJ) tree with Kimura-2-parameter and a (B) Maximum Likelihood method based on the nearest-neighbor interchange algorithm and general time-reversible (GTR) substitution model. The bootstrap values >65% from 500 replications are shown above the branches. Trees are drawn to scale, with branch lengths measured in the number of substitutions per site. 74 nucleotide sequences were included in the analysis. All positions containing gaps and missing data were eliminated.

The neighbor-joining (NJ) phylogeny derived from the concatenated loci is shown in Fig 1A. Most tested race 3 isolates fell into a single large clade with a high bootstrap value of 100 (dashed box A). However, race 3 isolates were observed in two other clades and some race 3 isolates displayed a weak aggressiveness phenotype. The Maximum likelihood [40] tree with the highest negative log likelihood score (15259.1007) derived by concatenated loci is in Fig 1B. The clades with high bootstrap values were similar for both NJ and ML trees. For instance, the two clades (dashed boxed B and C) of the NJ tree with 15 and 8 isolates, respectively, were represented in the ML tree as well.

The clusters of isolates showed a slight correlation with their sampling location. Several isolates from the same geographic subpopulation grouped together; however there were isolates such as those from Lee county (south subpopulation) that grouped close to the Madison county isolates (north subpopulation). Furthermore, when the *Fusarium* isolates were analyzed by AMOVA to test for the significance of population structure between populations, molecular variance was significant (*P* < 0.05) and accounted for 25.4% of the total variation (Table 5). This result indicates some evidence for the existence of genetic population structure based on geography. However, within population variation was much higher (F_ST_ = 0.75) suggesting that other factors may influence the genetic relationship between individual isolates.

**Table 5:** Pairwise comparison of Nei’s G_ST_ for loci BT, IGS, ITS, and TEF for *Fusarium* populations^a^.

	Pop1	Pop2
Pop2	0.012	
Pop3	0.046	0.058

^a^ *Fusarium* populations were grouped by origin of isolates in Florida: north (Pop1, n = 44), central (Pop2, n = 16), and south (Pop3, n = 11).

### Haplotype networks

**Fig 2:**
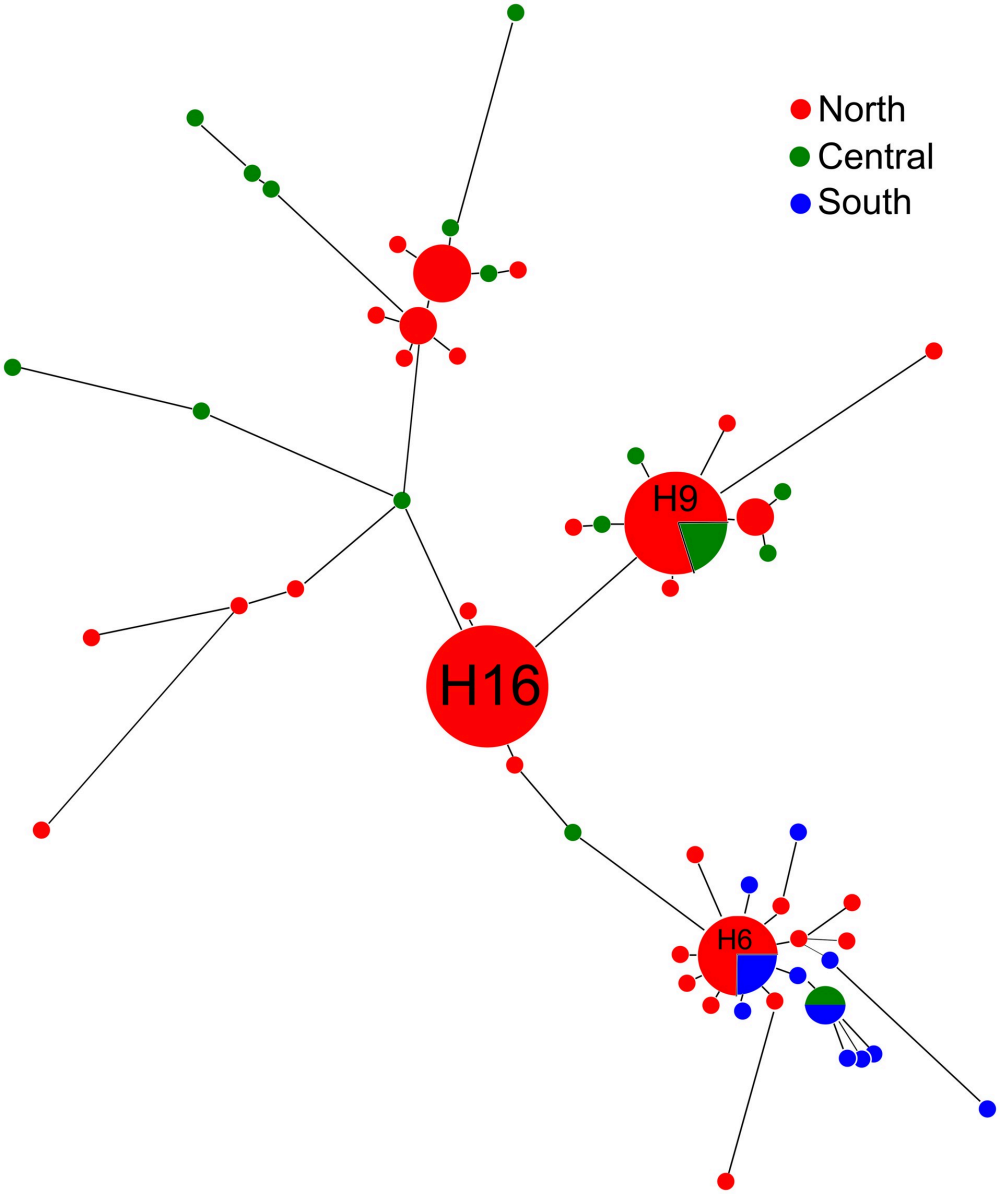
Haplotype network derived from concatenated sequencing data of BT, IGS, ITS, and TEF genes and loci and color-coded for *Fusarium oxysporum* f. sp. *niveum* subpopulations: North (Pop1, 44 isolates), central (Pop2, 16 isolates), and south (Pop3, 11 isolates). The connecting lines are proportional to number of mutations between isolates.

The population haplotype network for the tested isolates is shown in Fig 2. There were 55 haplotypes and the haplotype frequency, or the number of isolates represented by an individual haplotype, ranged from 1–6 with most haplotypes constituted from just a single isolate. Haplotypes with higher frequencies were mostly from the north population where the largest number of isolates were sampled (Pop1, 44 isolates). Haplotype 16 had the highest frequency with all isolates sampled from Alachua County north Florida (Pop1). Five isolates constituted the second most frequent haplotype, H9, including four isolates from Alachua County (Pop1) and Levy County, which belonged to the central region of Florida (Pop2). Haplotype H6 represented three isolates from Suwannee and Madison Counties (Pop1) and one isolate from Lee County in south Florida (Pop3). Pop1 had 31 haplotypes with 44 total isolates excluding the reference isolate. However, all isolates from Pop2 (n = 16) and Pop3 (n = 11) represented unique haplotypes. The haplotype network provided additional support for a correlation between geography and population genetic structure. The analysis suggested that the central (Pop2) and south Florida populations (Pop3) grouped farthest apart from each other while the north Florida population was a genetic intermediate. The pairwise comparison of G_ST_ also confirmed this (Table 5) by giving a higher distance between Pop2 and Pop3 populations than the other pairwise comparisons.

**Fig 3:**
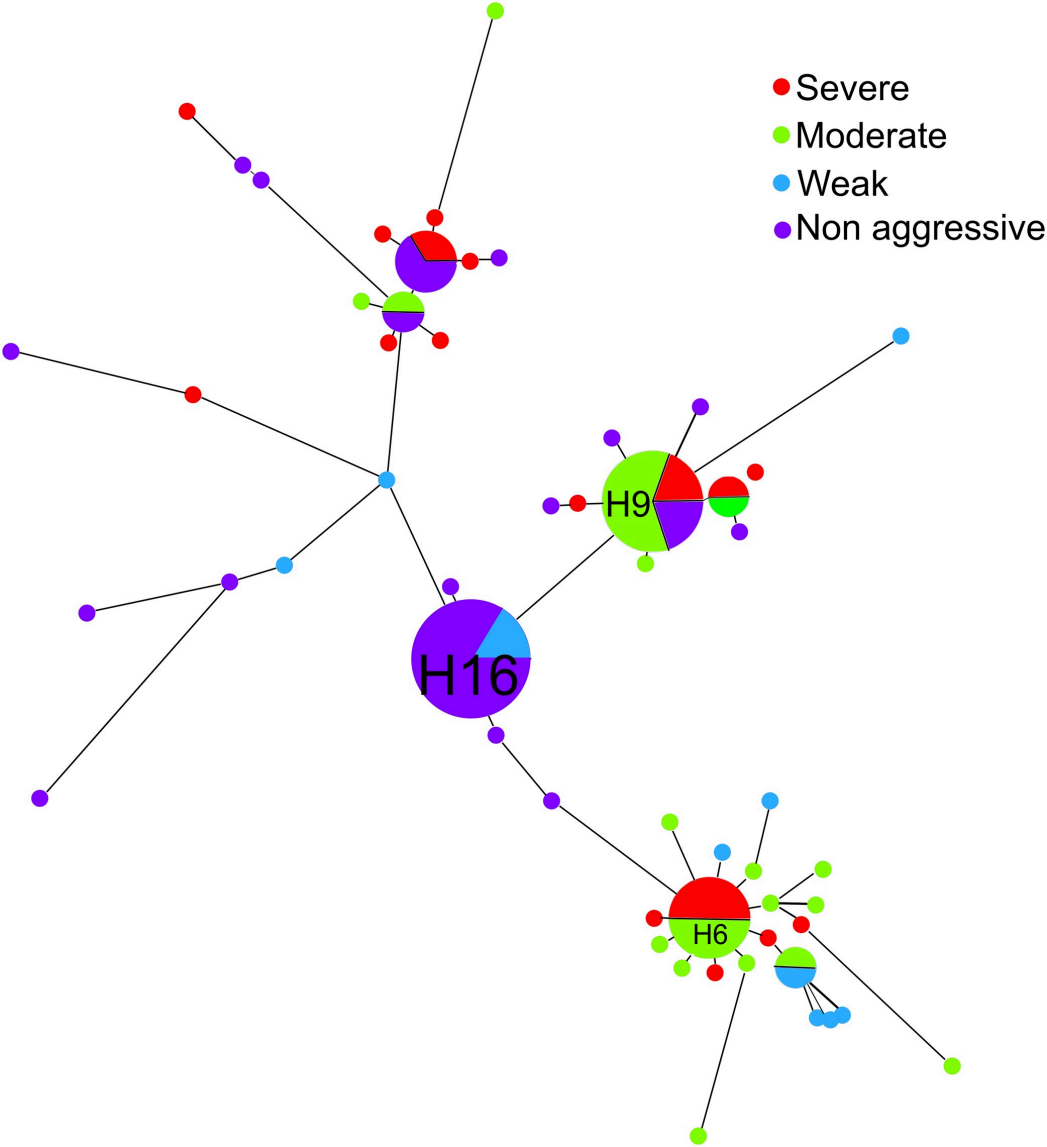
Haplotype network derived from concatenated sequencing data of BT, IGS, ITS, and TEF genes and loci and color-coded for level of aggressiveness (non-aggressive, weak, moderate, severe) of *Fusarium oxysporum* f. sp. *niveum* isolates. The connecting lines are proportional to number of mutations between isolates.

The aggressiveness of the isolates varied from non-aggressive to severe. The evenness of aggressiveness was a high 0.93 meaning that the relative abundance of the four levels of aggressiveness was not skewed amongst the isolates. Simpson’s index of Diversity (1-D) was 0.73 indicating considerable variation in aggressiveness among the isolates. Five (~6.94%) of the isolates were non-pathogenic, 24 (~33.3%) isolates presented a race 0 phenotype, 3 (4.16%) were race 1, 7 (~9.72%) showed a symptom profile consistent with race 2, 25 (~34.7%) isolates presented a race 3 phenotype, and 8 (~11.1%) were indeterminately identified as either race 2 or race 3. The amplification of the Six6 gene was positively correlated with either race 0 or 1, 83.33% of the time whereas the absence of this amplification was only positively correlated with 33.33% of race 2 isolates identified by bioassay. When the different categories of aggressiveness (non-aggressive to severe) were incorporated into the haplotype network there was no apparent effect on isolate grouping (Fig 3). Haplotypes with more than one isolate (H4, H5, H6, H9, H12, H15, and H16) always represented different categories of aggressiveness. For instance, H9 with five isolates fell into three aggressiveness levels: non-pathogenic, moderate and severe.

## Discussion

*Fusarium* isolates can be grouped according to their vegetative compatibility groups (VCG), formae speciales, and race types, which are primarily phenotypic groupings indicating genotypic traits. VCGs correspond to clonal lineages and isolates of the same VCG have the ability to form heterokaryons by anastomosis [65]. Individual *forma specialis* consist of isolates pathogenic to a particular plant host and each *forma specialis* can be comprised of one or more VCGs. *Formae speciales* can be further divided into race types based on their ability to infect one or more cultivar differentials. According to previous reports, the *Fusarium oxysporum* species complex (FOSC) is likely to consist of more than 80 plant host-specific *formae speciales* [66–68] and they have different relationships with VCGs and race types. Isolates of a single race may belong to several VCGs and a single VCG may include several race types [65]. Therefore, race types can have multiple origins or they could be closely related meaning one race type has the potential to evolve into a different race type. Molecular studies on some formae speciales with more than one VCG revealed polyphyletic relationships [27, 29, 33, 69]. For example, in a study by O’ Donnell, FOSC Fon isolates grouped in three clusters among other formae speciales showing polyphyletic evolution [33]. The numerous clusters with high bootstrap values exhibited by our isolates agree with this proposed polyphyletic origin for Fon.

Our MLSA revealed phylogenetic trees without clear organization based on race designation as several race 3 isolates were found outside the larger race 3 clade and interspersed amongst isolates of varying racial identity (Fig 1). The phylogenetic deposition of isolates with varying distinct race designations could have resulted from the origin of expanded pathogenicity beyond an isolate’s closer genetic relatives and independent of other race 3 isolates. This phenomena can occur during random mutations, transpositions and horizontal gene transfer events resulting in the *de novo* origin of VCGs and races [27, 33]. Conversely, there were isolates sampled from the same county and phylogenetically close but with distinct racial phenotypes. The observed differences in racial phenotype amongst otherwise genetically close relatives could indicate that race is influenced by a small subset of minor genomic changes such as single nucleotide polymorphisms; However, as we also observed isolates that shared the same race type and geography but were phylogenetically distant the possibility that structural variations in the genome contributes to racial phenotype cannot be ruled out.

This study revealed that Fon isolates collected from different regions of Florida represented different levels of aggressiveness spanning from non-pathogenic to severe. The four ad hoc aggressiveness levels: non-pathogenic, weak, moderate, and severe had a high evenness of 0.93 suggesting an even distribution of different levels of aggressiveness in the Florida Fon population. AMOVA showed there was a significant difference between *Fusarium* populations though within population variation was also high (75%). The pairwise distance of Pop1 and Pop2 was less than they were to Pop3. These results suggest that the Florida Fon population is diverse possibly as a result of the movement of isolates (e.g. human assisted) among regions and *de novo* evolutionary events within regions.

The haplotype network indicates signs of a central haplotype, H16, from which other haplotypes appear to have descended (Fig 2). H16 had a frequency of six and connected to two other closely related haplotypes each representing a single isolate. These eight isolates were grouped in a separate clade relatively distant from the other clades in the phylogram (Fig 1) possibly supporting an inferred ancestral sequence type. According to neutral coalescent theory, common or high-frequency haplotypes have more probability to be ancestral. Conversely, rare haplotypes are likely to be more recently evolved derivatives that differ by a few mutation events from their ancestors [70, 71]. These rare haplotypes tend to locate at the tip of the network and ancestral haplotypes are seen more centrally in the network [72]. Accordingly, some rare haplotypes in our network located close to high-frequency haplotypes showed a few mutations but not necessarily in a stepwise manner. Hypothetically, recently evolved haplotypes should be geographically closer to the ancestral haplotypes especially since Fon is a soilborne fungus with no known sexual stage. However, some rare haplotypes phylogenetically close to high-frequency haplotypes were geographically distant (Fig 2). This might be explained by human activities involving the movement of pathogen-contaminated seeds and planting material. Although we only recently published the first report of Fon race 3 in Florida [21], the present study shows race 3 has a widespread distribution, possibly as a result of human activity, or that race 3 is evolutionarily selected due to a competitive edge over other races. The recent emergence and quick dispersal of race 3 could be a result of recent changes in the implemented cultural farming practices of Florida farmers such as the widespread cultivation of susceptible seedless varieties and the removal of methyl bromide as a pre-season prophylaxis.

Our study shows that virulent race types are widely present in Florida and that local populations of Fon retain robust genetic diversity facilitating active adaption to disease management strategies. Further studies are needed to identify the genetic and molecular mechanisms determining an isolate’s pathogenicity and to support the development of efficient, high-throughput molecular identification assays which can replace the more laborious bioassay. While molecular markers have been reported from other countries [11, 73], they were not successful in identifying race 3. A thorough understanding of local Fon populations and their capacity to overcome deployed disease management strategies is necessary to support the state’s watermelon producers.

## Supporting information

S1 File Aggressiveness and race typing experimental results. (XLSX)

S1 Table NCBI accession numbers for sequenced genes and loci described in this research. (DOCX)
