# Supplementary material for: Phylogenetic and phenotypic characterization of Fusarium oxysporum f. sp. niveum isolates from Florida-grown watermelon
Source: PLoS One. 2021 Mar 25;16(3):e0248364. doi: 10.1371/journal.pone.0248364 (PMC7993609; doi:10.1371/journal.pone.0248364)
Supplement: S1 Table — (DOCX) [file pone.0248364.s002.docx]

**S1 Table**. Genbank accession numbers for tested isolates.

| **Sequence ID** | **TEF** | **BT** | **IGS** | **ITS** |
| --- | --- | --- | --- | --- |
| 150523 | MN418008 | MN437381 | MN428244 | MN423179 |
| 150514-1 | MT476636 | MN437389 | MN428284 | MN423205 |
| 150328 | MN418013 | MN437390 | MN428250 | MN423206 |
| 150601-5 | MN418014 | MN437391 | MN428285 | MN423207 |
| 150321 | MN418015 | MN437392 | MN428251 | MN423208 |
| 150319 | MF784505 | MN437393 | MF784502 | MN423186 |
| 150602-1 | MN418016 | MN437394 | MN428252 | MN423187 |
| 150601-4 | MN418017 | MN437395 | MN428286 | MN423209 |
| 150515-5 | MN418018 | MN437396 | MN428287 | MT460375 |
| 140507-b | MT476637 | MN437397 | MN428288 | MN423189 |
| 150515-2 | MN418009 | MN437382 | MN428245 | MN423180 |
| 110407.1-1.B2.F9C1 | MN418019 | MN437398 | MN428305 | MN423190 |
| 140507-a | MN418020 | MN437399 | MN428289 | MN423191 |
| 140508-b | MT476638 | MN437400 | MN428253 | MN423192 |
| 140411 | MT476639 | MN437401 | MN428254 | MN423193 |
| 150320 | MT476640 | MN437444 | MN428255 | MN423210 |
| 150514-3 | MN418010 | MN437383 | MN428281 | MN423181 |
| 150416 | MN418021 | MN437403 | MN428290 | MN423212 |
| 150524 | MN418022 | MN437404 | MN428257 | MN423213 |
| 130507 | MN418054 | MT408158 | MN428246 | MN423203 |
| 150525 | MF784504 | MN437405 | MF784503 | MN423214 |
| 150526 | MN418023 | MN437406 | MN428258 | MN423215 |
| 150527 | MT476654 | MN437407 | MN428259 | MN423216 |
| 150515-3 | MN418024 | MN437408 | MN428291 | MN423217 |
| 150516-2** | MT476642 | MN437409 | MN428292 | MN423218 |
| 150601-1 | MN418025 | MN437410 | MN428293 | MN423219 |
| 150601-2 | MN418026 | MN437411 | MN428260 | MN423220 |
| 150601-3 | MN418027 | MN437412 | MN428294 | MN423221 |
| 111006 | MN418028 | MN437413 | MN428261 | MN423222 |
| 111017 | MN418029 | MN437414 | MN428295 | MN423223 |
| 150514-2 | MT507286 | MN437384 | MN428247 | MN423204 |
| 140506 | MT476643 | MN437445 | MN428262 | MN423194 |
| 140704 | MN418030 | MN437415 | MN428304 | MN423224 |
| 150318 | MN418031 | MN437446 | MN428263 | MN423195 |
| 150322 | MT476644 | MN437447 | MN428264 | MN423225 |
| 150323 | MN418032 | MN437416 | MN428296 | MN423226 |
| 150324 | MN418033 | MN437417 | MN428265 | MN423227 |
| 150325 | MN418034 | MN437418 | MN428266 | MN423228 |
| 150326 | MT476645 | MN437419 | MN428297 | MN423229 |
| 150327 | MN418035 | MN437420 | MN428267 | MN423230 |
| 150329 | MN418036 | MN437421 | MN428268 | MN423231 |
| 150515-1 | MN418011 | MN437385 | MN428248 | MN423182 |
| 150330 | MN418037 | MN437422 | MN428269 | MN423232 |
| 150408 | MN418038 | MT408159 | MN428298 | MN423233 |
| 150409 | MN418039 | MN437423 | MN428270 | MN423234 |
| 150410 | MN418040 | MN437424 | MN428306 | MN423235 |
| 150411 | MT476646 | MN437425 | MN428299 | MN423236 |
| 150412 | MT476647 | MN437426 | MN428300 | MN423237 |
| 150413 | MN418041 | MN437427 | MN428271 | MN423238 |
| 150414 | MN418042 | MN437428 | MN428272 | MN423239 |
| 150417 | MN418043 | MN437448 | MN428301 | MN423240 |
| 150512 | MT476655 | MT408160 | MN428273 | MN423241 |
| 150515-4 | MN418012 | MN437386 | MN428282 | MN423183 |
| 150513 | MN418044 | MN437429 | MN428302 | MN423242 |
| 110407.1-1.B2 | MT476648 | MN437430 | MN428311 | MN423243 |
| 110407.1-1.B2.F9C2 | MN418045 | MN437431 | MN428312 | MN423244 |
| 110407.1-1.B2-F8 | MT476649 | MN437432 | MN428313 | MN423245 |
| 110407.1-1.B2-F8C | MT476650 | MN437433 | MN428307 | MN423246 |
| 110407.2-1 | MT476651 | MN437434 | MN428274 | MN423247 |
| 110407.3-1.1 | MN418046 | MN437436 | MN428275 | MN423248 |
| 130513 | MT476634 | MN437387 | MN428283 | MN423184 |
| 110407.3-1.2 | MT476652 | MN437437 | MN428309 | MN423249 |
| 110407.3-1.B | MN418047 | MN437438 | MN428314 | MN423250 |
| 110407.3-2.2 | MN418048 | MN437439 | MN428276 | MN423196 |
| 110407.3-4.B1 | MN418049 | MN437440 | MN428277 | MN423251 |
| 110407.3-4.B2 | MN418050 | MN437441 | MN428310 | MN423197 |
| 110407.3-4.B3 | MN418051 | MN437442 | MN428278 | MN423198 |
| 111018-2 | MT476653 | MN437449 | MN428279 | MN423199 |
| 111018-4 | MN418052 | MN437443 | MN428303 | MN423200 |
| 140508-a | MT476635 | MN437388 | MN428249 | MN423185 |
| 150516-1** | MN418053 | MN437450 | MN428280 | MN423201 |
| 150331* | MT476641 | MN437402 | MN428256 | MN423211 |
